# Introductory Lecture, Delivered before the Class of the Baltimore College of Dental Surgeons, at the Opening of Its First Session

**Published:** 1841

**Authors:** Chapin A. Harris

**Affiliations:** Professor of Practical Dentistry


### INTRODUCTORY LECTURE,

*Delivered before the Class of the Baltimore College of Dental Surgery, at the opening of its first Sesssion, by* CHAPIN A. HARRIS, M. D., D. D. S., *Professor of Practical Dentistry; furnished for the American Journal of Dental Science, at the request of the Publishing Committee.*

In entering upon the duties of the chair, to which I have been called, in the "Baltimore College of Dental Surgery," allow me to observe, that howsoever much of interest or curiosity the establishment of this institution—the first of the kind that has ever existed, either in this or any other country—may have awakened, it constitutes an era in the history of a most useful and valuable department of medicine; and if it be properly conducted, cannot be otherwise than productive of great good. Practiced as this branch of surgery too frequently has been, and in many instances even is at the present day, by individuals not having been specially educated for it, it is little better than a wide spread system of *quackery;* and that under such circumstances unfavourable results should often be experienced, is most reasonable to suppose. Few facilities have hitherto existed for obtaining the necessary preparatory instruction, and it has only been by unwearied industry, and patient and persevering toil, that those who have obtained respectability and usefulness in the profession, acquired the information that enabled them to do it. But of the number now engaged in its practice, few comparatively, have thought it necessary, or had the ambition to attempt to surmount the obstacles that presented to the acquisition of a thorough and scientific knowledge of the art. Rather than do this, the majority have been content to exercise its duties the best way they could—seeming to care little, whether the principles upon which they practiced were correct, or the services they rendered their patients of value or not, but depending more, for public notoriety and favour, upon artful management, than skill; they, as in too many instances has been the case, have sought the former to the neglect of the latter.

No credential or evidence of competency having been looked for or required, the profession has become crowded with individuals, ignorant alike of its theory and practice; and hence its character for respectability and usefulness has suffered in public estimation, and a reproach been brought upon it, which it would not otherwise have deserved.

The community at present is unable to discriminate between the well-educated and skilful practitioner, and the merest pretender—and until it shall be able to do this, more or less of the reproach that is brought upon the pursuit by the latter, will be visited upon the former. Often has the cheek of the honorable high-minded practitioner been caused to blush with shame on account of the mal-practice of the ignorant and unscrupulous of his professional brethren—often have his feelings suffered the deepest and most painful mortification at beholding injuries that had been inflicted on the teeth of individuals, who, in a spirit of the most entire confidence, had submitted them to the care of those not properly skilled in the treatment of the maladies of these organs.

Accessible as has been the calling of the dentist to all that were disposed to engage in it, and that too, without regard to qualification, it has been resorted to by the ignorant and illiterate, and, I am sorry to say, in too many instances, by unprincipled individuals, until it now numbers in the United States about twelve hundred, and of which, I think it may be safely asserted, not more than one-sixth, possess any just claims to a correct or thorough knowledge of the pursuit. That under such circumstances, it should occupy a place in the world's estimation, inferior to that to which it would otherwise be entitled, is not a subject of wonder.

Gladly would I draw a veil of secrecy over these things, but I feel myself bound in justice to the better-informed of the profession, to the public and to my own reputation, to denounce the empiricisms that have and do still exist in this department of medicine. In fact, so notorious have they become, that they are more or less familiar to all.

Of the qualifications necessary to be possessed by a dental practitioner, and the time required for their acquisition, few seem to be aware. On this subject an erroneous opinion seems pretty generally to prevail. A little mechanical tact, or dexterity, is thought by some, to be all that is requisite to a practitioner of dental surgery, and that this could be obtained in, at most, a few weeks. The prevalence of this belief, has given countenance to the assumption of the profession, by individuals totally disqualified to take upon themselves the exercise of its complicated and difficult duties. But it is to be hoped that the day is not remote, when it will be required of those to whom this department of surgery shall be entrusted, to be educated men, and well instructed in its theoretical and practical principles. Elevate the standard of the qualifications of the dental surgeon to a level with those of the medical practitioner, and the results of his practice will be always beneficial, which, at present are frequently the reverse. Require of the practitioner of dental surgery to be educated in the collateral sciences of anatomy and physiology, surgery, pathology and therapeutics, and the sphere of his usefulness and his respectability will be increased. Require of him to be thus qualified, and he will be able to contribute to the advancement and dignity of his calling, and by a zealous devotion to it, he will soon arrive at an excellence, to which, heretofore, but few comparatively, have attained, and enjoy the high gratification of knowing that he is a benefactor of his fellows.

Those who assume it without the necessary pre-requisite knowledge, ruin hundreds of teeth, before they are able, if indeed they ever are, properly to treat their diseases, and yet, that vast numbers do, every day furnishes the most indubitable evidence. Practice in this department of surgery, unless based upon a correct and thorough knowledge of its principles, can never be beneficial; but, on the contrary, will often increase the evil it is intended to remedy.

This being true, it would be well, were those who sought the services of the dentist, always to observe the caution given by Dr. Brown in his "Dentologia."

> "Beware of those whom science never taught
> The hard but useful drudgery of thought,"

for, it may be safely asserted, that those who have not energy of mind enough to think, or are unwilling to labour for information, can never arrive at much excellence in the pursuit, and are therefore, unworthy of confidence and patronage.

> "On just desert let all success attend,
> And patient merit never want a friend,"

adds, a little further on, the author from whom I have just quoted. But we might here pause, and ask,—Are confidence and patronage always extended to the deserving? Are the meritorious always prefered to the unmeritorious? Do those who have toiled for years to acquire a knowledge of the profession, that, when they should enter upon its duties, they might be useful to their fellow men, always receive a reward commensurate with their deserts? Proofs to the contrary might be adduced. But, although it often happens, that the ignorant pretender and the pompous charlatan, with their cunning and trickery, and self-urged pretensions, attract for a time the credulous multitude, their fame is rarely of long duration; and it is gratifying to know, that though the man of science and real worth, may be overlooked for a season, it is only he that acquires a reputation that is lasting and respectable.

To a successful practitioner of dental surgery, as I have on another occasion remarked, qualifications of a peculiar kind are requisite,—such as can only be acquired by study, and by witnessing the various and diversified manipulations that pertain to the several branches of the art, and by frequently repeating them under the immediate direction of a competent teacher. There is no branch of medicine or surgery, in which this kind of instruction is more essential than this. The reading of books that treat on the subjects necessary to be understood, though indispensably requisite, can never make an accomplished and skilful dental surgeon. The duties that devolve upon the dentist, will not admit of mediocrity in their exercise. It is therefore requisite that he be acquainted with such branches of knowledge as will enable him to perform them in the most perfect manner.

For example :—the plugging of a tooth is an operation which, unless well executed, instead of being beneficial, will generally accellerate the disease it is intended to arrest. Now, this operation, though purely mechanical, and thought by most persons to be easy of performance, requires a degree of skill, to which comparatively *very few* have been able to attain. That so small a number of those who have taken upon themselves the management of the teeth, should be capable of properly performing it, is greatly to be regretted ; for it is one of the most valuable operations that can be performed for the preservation of these organs.—When well performed, and with a suitable substance, it effectually arrests the progress of their decay in the parts of them in which the fillings are placed. If it fails to do this, it is owing to a want of skill on the part of the operator, or to some peculiarity of circumstances, under which the operation is performed. The practice, therefore, should not as is often done, be condemned because of its failure in the hands of individuals unskilled in the business, or the unfavourableness or peculiarity of circumstances under which it may have been performed. Its efficacy has been fully tested. Thousands have experienced the benefits resulting from it, and can testify in its favour. Its value being thus incontestibly established, it might seem unnecessary to dwell on the subject ; yet having incidently adverted to it, I would embrace the opportunity to refer briefly to a few of the causes of the want of success that has attended it ; and these may be stated in few words.

The first is, imperfect and improper manner of preparing the tooth previously to the introduction of the substance or substances, with which it is intended to be filled ; and here I would observe, that unless every particle of disordered bone be removed, and the cavity suitably shaped, however well the balance of the operation be performed, no very favourable or lasting good results can be expected. This part of the process requires not only judgement, but also considerable patience and tact.

The second is, imperfect manner of introducing the substance or substances employed, into the cavity of the tooth ; for, where the cavity is not so filled as to exclude every particle of moisture from the mouth from between the filling and surrounding bone, the progress of the decay, instead of being arrested, will generally be increased, and this is one of the most difficult parts of the operation. The filling to be serviceable, must be impenetrable to the fluids of the mouth, be flush with the surface of the tooth, and possess a high polish.

The third, and last which I propose to notice, is the employment of substances not capable of resisting the action of the fluids of the mouth. The use of such, has been very common, and has done much to bring the practice into disrepute. Gold and platina are the only substances employed for this purpose, capable of resisting the corrosive action of these secretions, when in a vitiated or impure state, and of these, the first is by far the best. There are circumstances, however, under which even tin and lead may be used with, not only impunity, but advantage ; but as these are liable to change, I do not consider their employment advisable under any. Gold may, therefore, be considered as preferable to every other substance ; for, if a tooth be worth filling at all, it is worth being filled in the very best manner, and with the very best material.

But, materials more objectionable than either tin or lead, have been used. An alloy composed of bismuth, tin, and lead, was a few years since, highly recommended. These metals, when combined in proper proportions, fuse at a very low heat; and it was in this state that the alloy was used, but its introduction into a tooth was found to be productive of great pain ; and besides that, when its temperature came down to that of the mouth, it did not fill the cavity—consequently its employment was soon abandoned.

More recently an *amalgam of mercury* and *silver*, has been highly extolled by a few practitioners, both in this and other countries; but by most of those who have had teeth filled with it, bitterly denounced—so that this, like the article last noticed, has nearly gone into disuse. It is certainly one of the most objectionable articles for filling teeth that can be employed, and yet from the wonderful virtues ascribed to this pernicious compound by those who used it, thousands were induced to try its efficacy.

The employment of this article, in the United States, for filling teeth, was first proposed, about five years since, by two celebrated European empirics, whose *harlequinade* in one of our northern cities, will not soon be forgotten by the hundreds, and I might say, thousands, that were attracted by it, and induced into the belief that the extravagant promises of these impudent impostors would be fulfilled. They however paid dear for their credulity, and many will have cause to remember them with sorrow and bitter execration to the latest period of their lives. But no sooner was the imposition discovered, than the indignation of those who had suffered by it was visited upon its authors with such effect that they were compelled to fly with their ill-gotten gain and blasted reputation, for refuge to another land.

Thus was the career of these celebrated mountebanks on this side of the water brought to a close, and it is to be hoped that the notoriety which they here gained may follow them wherever they go, that they may not again be able to impose, in a similar way, on the people of other countries.

With the route of these notorious impostors, it was thought that the use of this deleterious compound, for filling teeth, would be discontinued, but in this, expectation was disappointed. It was soon revived, though under different names from that by which it had before been designated, but so far as has come to my knowledge, the experiment in every instance has resulted in the injury of those who tried it.

Various other articles and preparations have, at different times, been recommended for filling teeth, but none have been found to answer so well as pure gold. In fact, there can be nothing better than this, nor indeed, is any thing better wanted, for when used by a skilful hand, it, in almost every instance, effectually prevents the further decay of the part of the tooth in which it is placed, and this is all that is required. Whenever, therefore, I hear of an individual's advertising that he has discovered or uses something better, I am induced to believe that he is either ignorant of this nice and difficult operation, or wishes to gain a notice to which his skill does not entitle him, and consequently cannot regard him as worthy of confidence. If he is really ignorant, he is not justifiable in asserting that which is not true, and if he is informed on the subject, he acts dishonestly in attempting to deceive, so that in either case he is culpable.

The treatment of superficial or incipient caries on the sides of the teeth that come in contact with each other, by its removal with a file, is a practice, that has been as much, if not more, abused, than that of any deep seated, by its removal and the filling of the cavity; and in consequence, has been thought to be of doubtful efficacy. In fact, it is regarded by very many, as productive of actual injury; but this is owing to its having been entrusted to the hands of individuals unacquainted with the manner in which the operation should be performed. In the filing, as in the plugging of teeth, judgment and skill are required. Unless it be judiciously and correctly done, it will often be followed by injurious instead of beneficial results. The skepticism, therefore, that exists, as to the good effects resulting from the use of the file upon the teeth, is not a matter of surprise.

That the operation does not necessarily cause the teeth upon which it is performed, to decay, has been conclusively evidenced. There are those around us, who have teeth that were filed, five, ten, and even twenty or thirty years ago, that are now perfectly sound. The value of the operation, notwithstanding the bad effects that have resulted from its unskilful performance, is well established. In the treatment of superficial caries, it is often invaluable.

Thus then it is seen, that even this, seemingly the simplest of all the operations of the dentist, cannot with safety be entrusted to one unskilled in the business. The mere passing of a file between the teeth is not all that is requisite. The manner of performing the operation, and the extent to which it should be carried, can only be properly determined by the nature and peculiarity of the decay, the age and temperament of the subject, and the physical structure and constitutional formation of the organs. With these the operator should be acquainted, to enable him to fulfil the indications of each individual case. Without this knowledge, he will be more apt to do injury than good. In short, the treatment of every disease to which the teeth are subject, unless conducted upon correct pathological principles, and by one well exercised in the manipulations pertaining thereto, will, in by far the greater number of instances, result unfavourably, and yet, there are no maladies incident to the body, that can with more certainty be subdued than those that are confided to the dentist.

Many of the diseases that come within his province, result from, or are influenced by an unhealthy action of some other part, and sometimes from a morbid diathesis of the whole organism, and to their successful treatment, reference to these, often becomes necessary. But, if the operator be ignorant of the anatomical structure of the body, and the laws of disease generally, he will be unable to pay that attention to them, which the exercise of the duties of his own particular department, often requires.

In view of these facts, for facts they are, which no one acquainted with the subject will hesitate to acknowledge, the importance of a more thorough and extensive knowledge, than is possessed by a large number of those now engaged in practice, must be very evident.

The preservation of the teeth is a matter of no trifling importance. Upon this depends, to a very considerable extent, the comfort and health of the body; their treatment then, when diseased, should only be submitted to such as are known to be "*au fait*" in the art.

But to proceed,—There is one other operation which, before I dismiss this part of the subject, it may be well to notice. It is the removal of that earthy substance (commonly called tartar) which forms more or less on the teeth of every one, but on some, in much greater abundance than others. In consequence of the imperfect manner in which this is frequently done, many are prejudiced against it. Finding that it re-accumulated, and that they had not derived the advantage from its removal which they had been led to expect, and that decay aftewards showed itself in their teeth that they had not before seen, not a few have been induced to believe, that the operation was not only productive of no advantage, but was attended with injury. Now this may be attributable to two causes. First, to the imperfect removal of the tartar from the teeth: Second, to the want of proper attention by the individuals themselves, to the use of the means necessary to prevent its re-formation.

If any particles be allowed to remain upon the teeth, they will act as nuclei around which it will regather, and besides, they will be productive of irritation to the gums, and prevent them from taking on a healthy action, so that no advantage will have been derived from its partial removal; and in those cases where there is a constitutional tendency of the general system, favouring the formation of this substance, constant attention to the cleanliness of the teeth is necessary to prevent its re-accumulation. In this case, the fault is not with the dentist, for however well he may have performed his duty, a re-deposition will soon take place, unless proper preventive means be frequently and regularly employed, and this can only be done by the patient. The opinion that its removal sometimes causes the teeth to decay, is erroneous. It may expose a decay that previously existed, but is never instrumental in its production. Its presence, by inflaming the gums, and thus causing the fluids of the mouth to become unhealthy, is often an indirect cause of decay to these organs.

Instead then of its removal being an injury, it is, when properly and effectually done, of incalculable advantage. By allowing it to remain upon the teeth, it not unfrequently accumulates to such an extent, as to give to the mouth a most disagreeable appearance, and to the teeth, it is productive of more injury than almost any other cause. Its effects upon the gums and fluids of the mouth, often result in the destruction of the alveoli and the loosening and loss of the teeth.

Besides the actual injury to which it gives rise, the beauty of the teeth is impaired and sometimes wholly destroyed by it; and the prevention of this alone, ought to constitute a sufficient motive for its removal, for there is nothing that contributes more to the agreeableness of the countenance, than a white and clear set of teeth. A beautiful denture constitutes a charm that is excelled only by a rich and well cultivated mind. Neither elegance of dress, nor costly jewels, can supply or compensate its want. These may dazzle the eye, but cannot command the admiration that is elicited by the other.

If then, white and clean teeth are desirable, and there is no one so much wanting in refinement and good taste, as to assert the contrary, the formation of this substance upon them, should never be permitted; but if from neglect, it shall have been allowed to accumulate, no time should be lost in having it removed. The importance of this cannot be too strongly urged. As has been before stated, it is essential to the health, to the appearance and to the preservation of the teeth, and besides, it is essential to the health of every part of the mouth, and to the whole organism.

The preservation of these organs, should be an object of solicitude with every one, and should constitute the first care of the dentist. It is in this that his skill is tested, and his services most valuable. The restoration of a diseased tooth to health, should be a source of much greater gratification to him, than the supplying of the loss of half a dozen with artificials. One good natural tooth, is worth more than a dozen that can be placed in the mouth by art; and yet, there are many who never think of consulting a dentist, except, perhaps, for the extraction of an aching tooth, until the desolating ravages of disease have made such inroads among their teeth as to render the substitution of these latter absolutely necessary. Artificial teeth, it is true, may be so inserted as to answer a most valuable purpose. If composed of proper materials, and applied upon correct principles, they may be worn with comfort, and subserve to a very considerable extent, the purposes of the natural organs; but they can never be made to equal them.

To prepare and insert artificial teeth so as to secure to the wearer all the benefits that can be derived from fictitious organs, though not requiring as much skill, as does the management or treatment of the diseases of the natural, is nevertheless exceedingly difficult, and can only be done by one well educated in the art. A man may be able to execute with accuracy and neatness, any piece of mechanism or apparatus, necessary to supply the loss of a single tooth, or even that of an entire denture, and still be unacquainted with the principles upon which it should be applied. There are no two cases alike, consequently, the rules that should govern the proceedure in one, will not be applicable in another. To a capacity to execute neatly the mechanical parts of the prosthesis, should be superadded, a knowledge of the physiological principles of the art.

Thus it may be seen, that the information necessary to be possessed, even in this department, is considerable; and that a *two weeks' studentship*, as some have supposed, is by no means sufficient for its acquirement.

This branch of the art, however, has arrived at a very high state of perfection. Artificial teeth are now made so as to resemble the natural organs so closely, that when properly inserted, few can discover wherein they differ ; and the advantages that are derived from them, when judiciously applied, are such that persons having once had them, are afterwards unwilling to do without them. It is desirable then, that those who occupy themselves with the prosthesis of these organs, be qualified to effect it in a manner that will secure to those who are so unfortunate as to require artificial teeth, every benefit that can be derived from such substitutes. Indeed, of so frequent occurrence is the loss of the natural teeth, and so much does that of a single one, particularly if it be in the front part of the mouth, impair the beauty of the countenance, and the articulation of the voice, that a resort to the dental surgeon, for artificial teeth, is almost as frequent, as that for the cure of the diseases of the natural ones.

This branch of the art is very ancient. It was practiced in Rome more than two thousand years ago ; but to what perfection cannot well be determined, since all the information that can be gleaned upon the subject, is derived from writers of antiquity, whose province admitted not of any thing more than casual references to it. From them, however, is learned, that it was practiced at that epoch, and that the artificial teeth there used, were formed from bone and ivory. It has only been however, until within the last thirty or forty years, that the prosthesis of the teeth has been very well understood, and the improvements of the last ten or fifteen have probably been greater than those of any other former period. During this time the art has been so much advanced, that it would seem almost impossible to bring it to a much greater state of perfection. Improvements, however, will doubtless continue to be made, both in this and every other branch of the profession. In fact, the efforts that are at this time being made, particularly in this country, by the more reputable of its members, to advance the science, cannot but be fraught with highly beneficial results.

Hitherto, individual exertions, have been relied upon for the advancement of the science, and it is to the efforts of a few, who have devoted themselves to its culture, with a perseverance and zeal, worthy of the highest praise, and more general imitation, that it has arrived at its present state of perfection. But, a new spirit of inquiry is awakened,—combined efforts are being made, to elicit additional light, and disseminate more thorough and general information on the subject.

But although, among the practitioners of dental surgery, there are very many possessed of talents and education, and who, by dint of unwearied industry, have acquired distinguished, and justly-deserved high reputations for skill; yet they, by their individual exertions, have been unable to free the profession from the reproach that has been brought upon it, by the hundreds of ignorant and disqualified individuals, by whom its duties have been and are still exercised. Encouraged, however, by what has been done by associated influence, and free interchange of sentiment, among the practitioners of medicine and surgery, they are beginning to lay aside that selfish ambition, and petty jealousy, which in times past have kept them apart, and are uniting their efforts to raise the standard of professional qualification—hoping thereby to secure for their vocation a confidence and a respectability that it has not hitherto enjoyed. That they may be successful in so commendable an undertaking, should be the ardent desire of every one; yet filled as the ranks of the profession are, with individuals who have never learned the first rudiments of the science, it will doubtless require some time to effect the wished reformation, and will only be accomplished, as they shall be able to fix a line of distinction between the competent and the incompetent. With a view to this and the more rapid improvement of the art, an association has recently been formed in this country under the designation of the "*American Society of Dental Surgeons*," with such terms of membership as will admit none, except such as are thoroughly qualified to practice the profession. If this feature be carried out, and from its present organization, no fears need be apprehended on the subject; the association will be able to furnish to its members, credentials of competency, that will, at once, command confidence and respect, and at the same time tend to secure the community against the impositions of the unprincipled charlatan and ignorant pretender. In addition to this, it will contribute in an eminent degree, to establish correct and uniform systems of practice throughout the whole country, and thus an almost incalculable amount of good may result from it.

But to Maryland belongs the honor and credit of establishing the first institution that has ever existed, for the education of gentlemen for this particular department of medicine. The Legislature of this State at its last session incorporated a College for this special purpose, and thus, facilities are offered for the acquirement of a knowledge of all the branches of this most valuable art, that have never before existed, and in consequence of which, the majority of those who have assumed its practice, have been compelled to do so without the requisite preparatory qualifications. Had facilities for obtaining the necessary information existed, it is probable that most of those who have thus entered the profession, would have availed themselves of them ; but as there were none, they were obliged to depend upon such instruction as they could procure, but relying principally upon what they should learn from practice. Thus, from year to year have numbers been added to the profession, until its ranks have become crowded with individuals totally disqualified to take upon themselves the exercise of its nice and critical duties.

But a new era has commenced in this department of knowledge, and I am flattered with the belief, that the time is not distant, when it will be expected of the dental surgeon, as it now is of the medical practitioner, that he shall have been regularly educated for the pursuit, before he shall be permitted to engage in it. For a neglect of this, no good excuse can hereafter be offered. The establishment of the institution to which I have just referred, affords opportunity for obtaining instruction in all the branches requisite to an accomplished practitioner ; and it may not be amiss to observe, that, possessed of ample means for doing this, it is the determination of those to whom its duties have been committed, to extend facilities for their acquirement, such as have never before been presented. Aware of the responsibility that rests upon them, the faculty will spare no efforts to make it creditable to the state that created it, and beneficial to the public. Conscious that its claims to respectability and usefulness will depend upon the manner in which they shall discharge their duties, it will be their constant endeavour to impart, not only correct, but thorough theoretical and practical information,—persuaded that without this, it is impossible for any to practice the art with credit to themselves, or for the benefit of their employers, they are resolved to admit none to the honors of the institution except such as possess it. In short, they are determined that no reproach shall rest upon them for fixing a standard of qualification, that shall not at once be respectable, and entitle those coming up to it, to the confidence of an enlightened community. Whether their efforts will be sustained in a manner to justify their continuance, time only can determine. The importance of a knowledge of the subjects proposed to be taught by them, to the dental practioner, would at least, seem to justify an affirmative conclusion, and that their labours will ultimately be crowned with success, they cannot but cherish a confident belief.

That a school, established for the special purpose of teaching a profession whose resources are as frequently called into requisition as those of the dental surgeon, and upon the judicious and correct exercise of whose duties, the comfort and happiness of so many depend, should be suffered to go down, I can hardly suppose. A failure of the experiment, for as yet, it can be regarded as little else, would be a reproach upon the intelligence of our country. Therefore, that it will be sustained, I entertain no scruples. The announcement that was made of its establishment by the Board of Visitors, soon after its organization, was received, by the more intelligent of the profession throughout the United States, in a manner calculated, not only to inspire its friends with confidence, but also that promises perpetuity to it. That it will have many discouragements and difficulties to contend with, is to be expected ; yet, if by the persevering exertions of those to whom its management has been entrusted, they be eventually overcome, and the objects for which it was created accomplished, they will feel themselves amply rewarded, for the time and labour which they shall have bestowed upon it.

For the friendly feeling that has been manifested towards it by most of the medical faculty of this city, as well as by gentlemen of that profession in other places, and for the flattering notices that have been taken of it by many of the medical and newspaper presses of the country, they not only feel profoundly grateful, but are stimulated by them, to enter upon its duties, with a zeal, which, perhaps they might not otherwise have possessed. And, in entering thus upon their labours, they indulge the hope, that the expectations of its friends will not be altogether disappointed.

In regard to the plan of instruction which it is proposed to pursue, it is only necessary to observe, that, while it will be such as will enable the pupil to become thoroughly grounded in theory, the surgical and mechanical portions of the art will be exhibited in all their minutiæ, and opportunity given to each, of repeating them sufficiently often to become familiarized with, and skilled in the various manipulations pertaining thereto, so that on leaving the school he may not only be a *good theorist*, but a *good practitioner.*

In conclusion, I would address a few words to the gentlemen of the class,—hoping that what I shall say, may not be without some profit to them.

Gentlemen,—the profession for which you are preparing, is honourable; it is useful; it is one that will enable you to be serviceable to your fellows,—to relieve much of human pain, and to mitigate many mortal woes. Though it may not require of you to combat the more violent and aggravated forms of disease that are incident to man, you will have to treat those that demand nearly or quite an equal degree of acumen and skill; and if you would possess these, it will be necessary that you devote yourselves diligently and perseveringly to their acquisition; that you faithfully employ the time that you design appropriating to your studentship, in acquainting yourselves with the structure of the parts implicated in them—their pathology and therapeutical indications. If you would be able to arrest the diseases that come within the province of the profession for which you are preparing, and prevent the various evils that are frequently consequent thereupon, or, in other words, if you would be able to preserve those invaluable organs, the teeth, and thus secure to those by whom you may be consulted, one of the greatest of earthly blessings, a healthy denture, endeavour to attain the knowledge that will enable you to do it.

If you would command respect, and enjoy the confidence of those among whom it may be your lot hereafter respectively to reside, let it be your persevering endeavour to deserve them. Resolve that you will not be satisfied with mere mediocral abilities in the calling for which you are qualifying. Resolve to put forth all your energies for its acquisition, that you may be able to take rank among the most scientific and skilful. If you do not do this, the fault will be your own, and I am sure that your teachers will labour to little purpose in endeavoring to impart instruction to you, unless you strive to profit therefrom. Let one then, who will feel a deep and anxious solicitude for your success, urge you to diligence in the pursuit of the knowledge you are now seeking. Its possession can alone enable you to discharge in a correct and proper manner, the intricate and complicated duties you are preparing to assume. Let then, a pure and high ambition stimulate you to zealous and unwearied exertion, and be assured that you will never be reproached by the future, for time misspent, or for failing to realize to those who shall seek professional aid at your hands, the benefits which the art is capable of bestowing.

